# Analysis of the Recovery of Immune Status in the Patients With Paroxysmal Nocturnal Hemoglobinuria After Treatment With C5 Complement Inhibitor

**DOI:** 10.1002/jcla.70211

**Published:** 2026-04-29

**Authors:** Hui Liu, Mengting Che, Liyan Li, Honglei Wang, Chaomeng Wang, Junshu Wu, Yingying Chen, Zhaoyun Liu, Lijuan Li, Rong Fu

**Affiliations:** ^1^ Department of Hematology Tianjin Medical University General Hospital Tianjin People's Republic of China; ^2^ Tianjin Key Laboratory of Bone Marrow Failure and Malignant Hemopoietic Clone Control Tianjin People's Republic of China; ^3^ Tianjin Institute of Hematology Tianjin People's Republic of China

**Keywords:** B lymphocytes, C5 complement inhibitor, immune escape, paroxysmal nocturnal hemoglobinuria, T lymphocytes

## Abstract

**Background:**

Currently, C5 complement inhibitors serve as the first‐line therapy for classic paroxysmal nocturnal hemoglobinuria (PNH), effectively controlling hemolysis and enhancing patients' quality of life. However, the impact of this treatment on patients' immune status remains unclear.

**Methods:**

A retrospective analysis was conducted on 27 PNH patients treated with C5 complement inhibitors, focusing on the dynamic changes and recovery of key immune cell subsets, including T lymphocytes, B lymphocytes, natural killer (NK) cells, and dendritic cells (DCs).

**Results:**

Among the 27 patients, 70.3% (19/27) achieved transfusion independence. Treatment responses were categorized as follows: complete response (7.4%, 2/27), major response (3.7%, 1/27), good response (25.9%, 7/27), partial response (22.2%, 6/27), minor response (29.6%, 8/27), and non‐response (11.1%, 3/27). Before treatment, the proportion of CD4^+^ T lymphocytes was significantly lower in PNH patients than in normal controls [(38.96 ± 8.270)% vs. (48.57 ± 5.760)%, *p* < 0.001], and it gradually recovered toward normal levels at 12 months [(42.48 ± 5.623)%] and 24 months [(43.71 ± 6.204)%] post‐treatment. In contrast, the percentage of CD8^+^ T lymphocytes was markedly elevated in pre‐treatment patients compared with controls [(49.38 ± 7.259)% vs. (41.75 ± 4.744)%, *p* < 0.01], and it returned to near‐normal levels at 12 months [(42.97 ± 7.074)%]. The proportion of B lymphocytes was significantly reduced in pre‐treatment PNH patients [(6.535 ± 1.854)% vs. (12.70 ± 2.256)% in controls, *p* < 0.001] and partially recovered at 6, 12, and 24 months post‐treatment [(8.266 ± 1.756)%, (8.626 ± 1.649)%, and (8.559 ± 1.697)% respectively], though it did not reach normal levels. No significant changes were observed in Treg cells, NK cells, and DCs after treatment.

**Conclusions:**

This is the first study to systematically analyze immune status alterations following C5 complement inhibitor treatment in PNH patients, suggesting that the complement system may be involved in the development of immune escape mechanisms in PNH.

## Graphical abstract

We retrospectively evaluated the impact of C5 complement inhibition on immune cell profiles and recovery in 27 PNH patients; both CD4+ and CD8+ T cells exhibited partial recovery, while B cell counts increased but remained below normal levels. In contrast, Treg cells were significantly reduced in PNH patients and showed no notable change post‐treatment. Neither NK cells nor DCs demonstrated significant recovery.

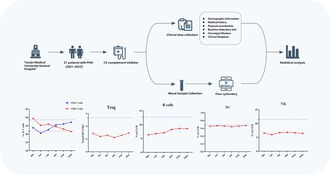

## 1 Introduce

Paroxysmal nocturnal hemoglobinuria (PNH) is a rare acquired hemolytic disease caused by somatic mutations of the phosphatidylinositol glycan‐A (PIG‐A) gene in the hematopoietic stem cell [1, 2]. PNH clones appear after PIG‐A gene mutation, and other factors are needed to give PNH clones a proliferation advantage, which together lead to the onset of PNH. Immune evasion plays an important role in PNH clonal proliferation [3, 4]. Complement C5 is at the end of the complement cascade reaction and is the most classic target for drugs inhibiting the complement pathway in PNH. It can effectively block intravascular hemolysis and reduce the need for blood transfusions [5, 6, 7]. It can also significantly reduce the incidence of thrombosis in PNH patients and improve the quality of life and the quality of patients and overall survival rate [8, 9]. It is a first‐line treatment option for classic PNH patients.

However, the effect of complement C5 inhibitors on the immune function of PNH patients has not been reported. This study retrospectively analyzed the clinical data of 27 PNH patients treated with complement C5 inhibitors, and the changes in the number of peripheral immune subpopulations before and after treatment, to explore the effects of complement C5 inhibitors on the immune function of PNH patients. The role of immune function in PNH patients and preliminary exploration of the possible clinical value of C5 complement inhibitors in the immune recovery of PNH patients.

## 2 Methods

### 2.1 Patients

All patients were diagnosed with PNH and received complement inhibitor therapy at the Department of Hematology, Tianjin Medical University General Hospital during the study period from January 2021 to December 2023. The diagnosis of all patients met the criteria of the International PNH Working Group and the “Guidelines for the diagnosis and management of paroxysmal nocturnal hemoglobinuria (2024)” [10, 11]. Among the 27 PNH patients included in this study, 22 received crovalimab as participants in a therapeutic clinical trial (ClinicalTrials.gov ID: NCT04654468). The remaining 5 patients received eculizumab as part of routine clinical practice.

The healthy control group comprised 30 age‐ and sex‐matched volunteers (16 males, 14 females; median age 33 years [range: 22–48]) recruited from the Health Examination Center of Tianjin Medical University General Hospital. To ensure a robust baseline, the inclusion criteria for controls were: (1) no history of hematological diseases, autoimmune disorders, infectious diseases, or malignant tumors; (2) normal results for routine blood analysis, liver and kidney function, and complement system evaluations; and (3) no use of immunosuppressive agents within the preceding 3 months. Controls were matched to the patient cohort by age (±5 years) and sex to minimize potential confounding variables.

The study protocol was approved by the Ethics Committee of Tianjin Medical University General Hospital.

### 2.2 Clinical Data Collection

Clinical data were collected prospectively and included demographic characteristics, medical history, physical examination findings, baseline laboratory parameters—hemoglobin (HGB), white blood cell count (WBC), absolute neutrophil count (ANC), reticulocyte count (Ret), lactate dehydrogenase (LDH), total bilirubin (TBIL), PNH clone size, plasma‐free hemoglobin, haptoglobin levels—and details of complement inhibitor therapy.

### 2.3 Complement Inhibitor Treatment Regimens

All patients received *Neisseria meningitidis* vaccination and complement inhibitor therapy was started at least 2 weeks later. Crovalimab 1000 mg, intravenous infusion on the first day, 340 mg subcutaneous injection on the second day at week 1, 340 mg subcutaneous injection on week 2, week 3, week 4, 680 mg subcutaneous injection from week 5, once every 4 weeks. Eculizumab 600 mg was administered once a week for 1 month, and 900 mg was administered intravenously every 2 weeks from the second month.

### 2.4 Clinical Response Assessment

The clinical response categories used in this study were based on the standardized efficacy evaluation criteria for PNH patients receiving complement inhibitors, as reported in the Chinese Guidelines for the Diagnosis and Management of Paroxysmal Nocturnal Hemoglobinuria (2024) [11].

### 2.5 Flow Cytometry

Cells were stained with cell surface markers for 15 min, washed with PBS, and resuspended again. For intracellular antibodies (BioLegend, California, U.S.), perform staining after permeabilizing the cell membrane. Cells were detected using BECKMANCOULTER instrument. Flowjo software were used to analyze.

### 2.6 Statistical Analysis

Statistical analysis was performed using SPSS version 23. Quantitative data were tested for normality, and mean ± standard deviation was used for statistical description when they obeyed normal distribution; the *t*‐test of related samples was used for comparison before and after treatment. *p* < 0.05 was considered statistically significant.

## 3 Results

### 3.1 Baseline Characteristics of Enrolled Patients

Of the 27 patients with PNH, 15 were male and 12 were female, with a median age of 35 (20, 50) years, including 23 patients with classic PNH and 4 patients with PNH accompanied by bone marrow failure; 22 cases were treated with Crovalimab, and 5 cases were treated with Eculizumab. The CD59‐erythrocyte ratio was (57.13 ± 21.34)%, the CD59‐granulocyte ratio was (69.57 ± 25.45)%, the CD14‐FLAER‐ ratio was (83.34 ± 23.21)%, and the CD24‐FLAER‐ ratio was (75.74 ± 28.34)%.

Among the 27 PNH patients treated with complement inhibitors, 70.3% (19/27) were free from blood transfusion, 7.4% (2/27) achieved complete response, 3.7% (1/27) had a major response, 25.9% (7/27) showed a good response, 22.2% (6/27) exhibited a partial response, 29.6% (8/27) demonstrated a minor response, and 11.1% (3/27) were non‐responders. The other baseline characteristics of the patients are shown in Table 1.

**TABLE 1:** Clinical baseline information of patients.

Clinical characteristics	Index
Age (median)	35 (20–50)
Gender	
Male	15
Female	12
Clinical classification of PNH	
Classic PNH	23
Combined with other bone marrow failure diseases	4
History of red blood cell transfusion in the past year	22
History of thrombosis	6
Baseline medication	
Anticoagulants	2
Steroid	27
Immunosuppressive therapy	6
C5 complement inhibitor therapy	
Eculizumab	5
Crovalimab	22
Ret(%)	8.70 ± 5.47
WBC (×10^9^/L)	4.21 ± 2.06
HGB (g/L)	79.32 ± 21.34
PLT (×10^9^/L)	129.23 ± 71.25
LDH (U/L)	1398.95 ± 712.43
Free hemoglobin (mg/L)	164.12 ± 89.97
CD59 − erythrocytes (%)	57.13 ± 21.34
CD59 − granulocytes (%)	69.57 ± 25.45
CD14 − FLAER‐ (%)	83.34 ± 23.21
CD24 − FLAER‐ (%)	75.74 ± 28.34
Effects (6 m)	
Complete response	7.4% (2/27)
Major response	3.7% (1/27)
Good response	25.9% (7/27)
Partial response	22.2% (6/27)
Minor response	29.6% (8/27)
Non‐responders	11.1% (3/27)

Abbreviations: HGB, hemoglobin; LDH, lactic dehydrogenase; PLT, platelet; PNH, Paroxysmal nocturnal hemoglobinuria; Ret, reticulocyte; WBC, white blood cells.

### 3.2 Both CD4+ and CD8+ T Cells Showed Some Recovery After Treatment With Complement Inhibitors

**FIGURE 1:**
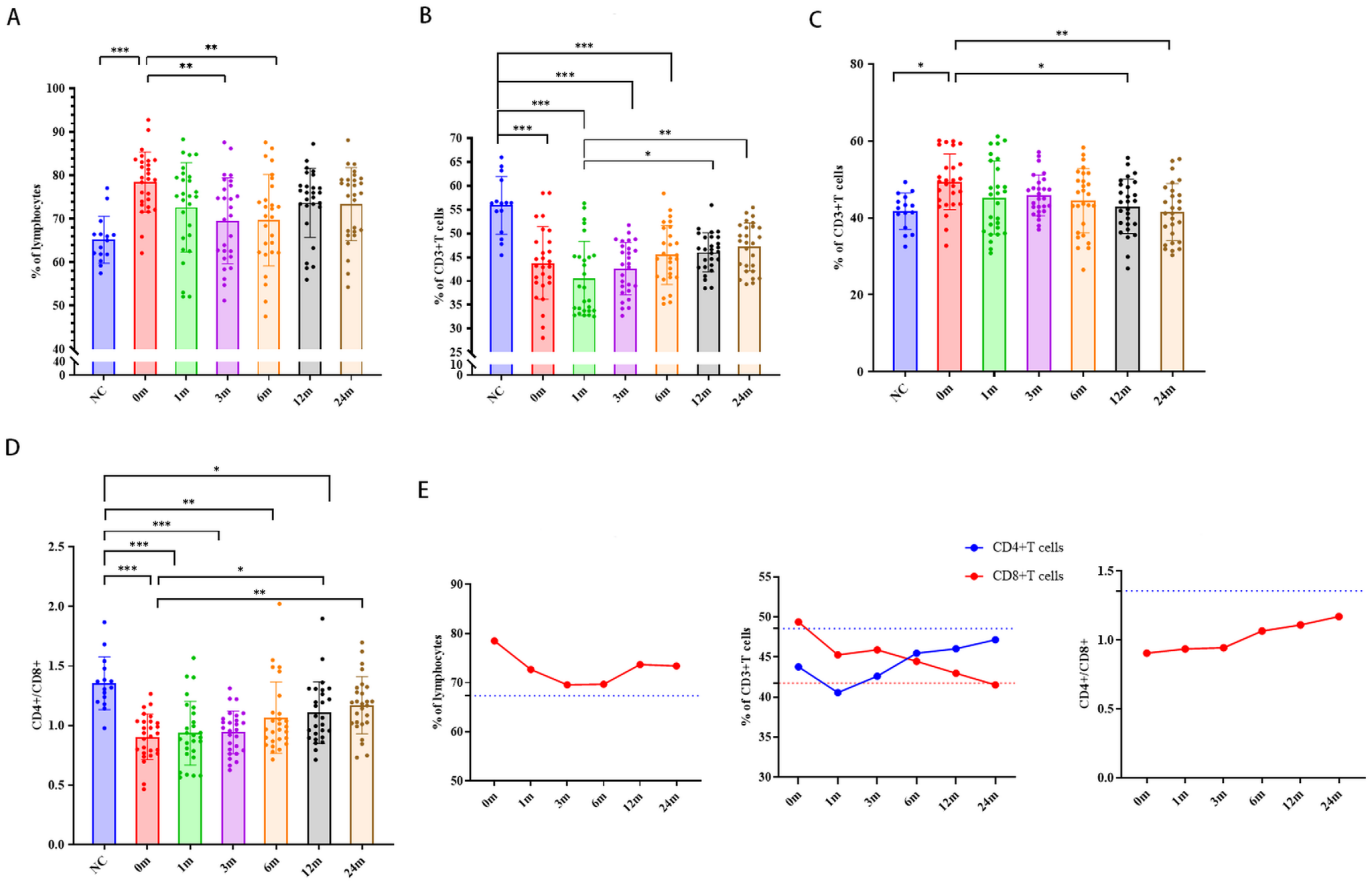
Changes in T lymphocyte subsets in PNH patients receiving C5 complement inhibitor therapy. (A) Total T cells, (B) CD4^+^ T cells, (C) CD8^+^ T cells, (D) CD4/CD8 ratio, and (E) dynamic changes of T lymphocyte subsets from baseline to 24 months of treatment. Data are presented as mean ± SD. *p < 0.05, **p < 0.01, ***p < 0.001.

The proportion of peripheral circulating T cells/lymphocytes in PNH patients was (78.52 ± 6.937)%, which was significantly higher than that in the control group (67.33 ± 5.025)% (p < 0.001). The number of T cells after treatment with complement C5 inhibitor decreased significantly at 1, 3, and 6 months, but showed an upward trend at 12 and 24 months (Figure 1A).

Further observation of the changes in T cell subsets revealed that the CD4^+^/CD3^+^ T cell ratio before treatment was (38.96 ± 8.270)%, which was lower than that in the control group (48.57 ± 5.760)% (*p* < 0.001); it remained at a low level in the first month after treatment and then gradually increased from 3 months, remaining significantly lower than normal levels at 6 months (*p* < 0.001); however, there was no significant difference at 12 and 24 months (Figure 1B).

The proportion of CD8^+^/CD3^+^ T cells before treatment was (49.38 ± 7.259)%, which was higher than that of the control group (41.75 ± 4.744)% (p < 0.01). After treatment, it showed a downward trend and was significantly lower at 12 and 24 months than that before treatment (Figure 1C). The change of the CD4/CD8 ratio was the most pronounced before and after treatment. Before treatment, it was significantly lower than the normal control, but gradually recovered after treatment. It had significantly recovered compared with the baseline (*p* < 0.05) at 12 months, although there was still a statistical difference with the normal control. At 24 months, it continued to rise compared to the baseline (*p* < 0.01), closing to the normal (Figure 1D, Table 2). The dynamic changes of T lymphocyte subsets from baseline to 24 months of treatment are shown in Figure 1E.

**TABLE 2:** Peripheral blood cell subsets in the patients with PNH.

Index	NC	0 m	1 m	3 m	6 m	12 m	24 m
T%	67.33 ± 5.025	78.52 ± 6.937a	72.69 ± 10.31	69.58 ± 9.907e	69.69 ± 10.52e	73.70 ± 7.970	73.43 ± 8.412
CD4 + T%	48.57 ± 5.760	38.96 ± 8.270a	40.55 ± 7.780a	38.00 ± 6.639a	40.25 ± 7.868a	42.48 ± 5.623i	43.71 ± 6.204h
CD8 + T%	41.75 ± 4.744	49.38 ± 7.259c	45.27 ± 9.513	45.89 ± 5.280	44.46 ± 8.368	42.97 ± 7.074f	41.52 ± 7.415e
CD4+/CD8+	1.36 ± 0.222	0.90 ± 0.191a	0.94 ± 0.2671a	0.9431 ± 0.1795a	1.066 ± 0.2988b	1.109 ± 0.2594cf	1.171 ± 0.2394e
Treg%	5.35 ± 0.697	2.88 ± 0.862a	2.38 ± 0.691a	2.60 ± 0.887a	2.24 ± 0.710a	2.57 ± 0.848a	2.95 ± 0.792a
B%	12.70 ± 2.256	6.355 ± 1.854a	6.746 ± 1.404a	7.060 ± 1.452a	8.266 ± 1.756afh	8.626 ± 1.649aegi	8.559 ± 1.697adgi
NK%	11.49 ± 2.500	6.535 ± 2.692a	6.009 ± 2.678a	6.694 ± 2.961a	6.784 ± 3.056a	6.628 ± 3.078a	6.413 ± 2.509a
DC%	0.68 ± 0.228	0.65 ± 0.255	0.67 ± 0.267	0.66 ± 0.234	0.64 ± 0.198	0.66 ± 0.175	0.67 ± 0.224

*Note:* Compared to NC group, a:*p* < 0.001; b:*p* < 0.01; c:*p* < 0.05; Compared to PNH group before treatment with C5 complement inhibitor, d:*p* < 0.001; e: *p* < 0.01; f:*p* < 0.05; Compared to PNH group at 1 month after treatment with C5 complement inhibitor, g:*p* < 0.01; h:*p* < 0.05; Compared to PNH group at 3 month after treatment with C5 complement inhibitor, i:*p* < 0.05.

### 3.3 Treg Cells Are Significantly Reduced in PNH, With no Significant Change After Treatment

**FIGURE 2:**
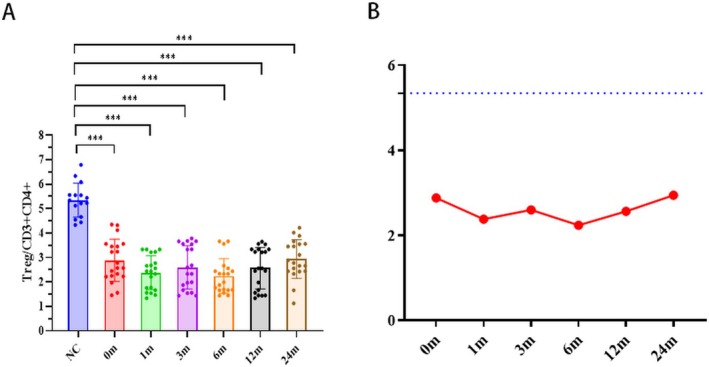
Proportions of Treg cells in PNH patients receiving C5 complement inhibitor therapy. (A) Treg cell proportions and (B) dynamic changes of Treg cells from baseline to 24 months of treatment. Data are presented as mean ± SD. *p < 0.05, **p < 0.01, ***p < 0.001.

The proportion of peripheral circulating Treg cells in PNH patients (2.884 ± 0.8624)% was significantly lower than that in the control group (5.347 ± 0.6970)% (*p* < 0.001). There was no statistically significant difference at 1 month, 3 months, 6 months, 12 months, and 24 months after complement C5 inhibitor treatment (Figure 2, Table 2).

### 3.4 B Cells Increased After Treatment, While Remained Below the Normal Control

**FIGURE 3:**
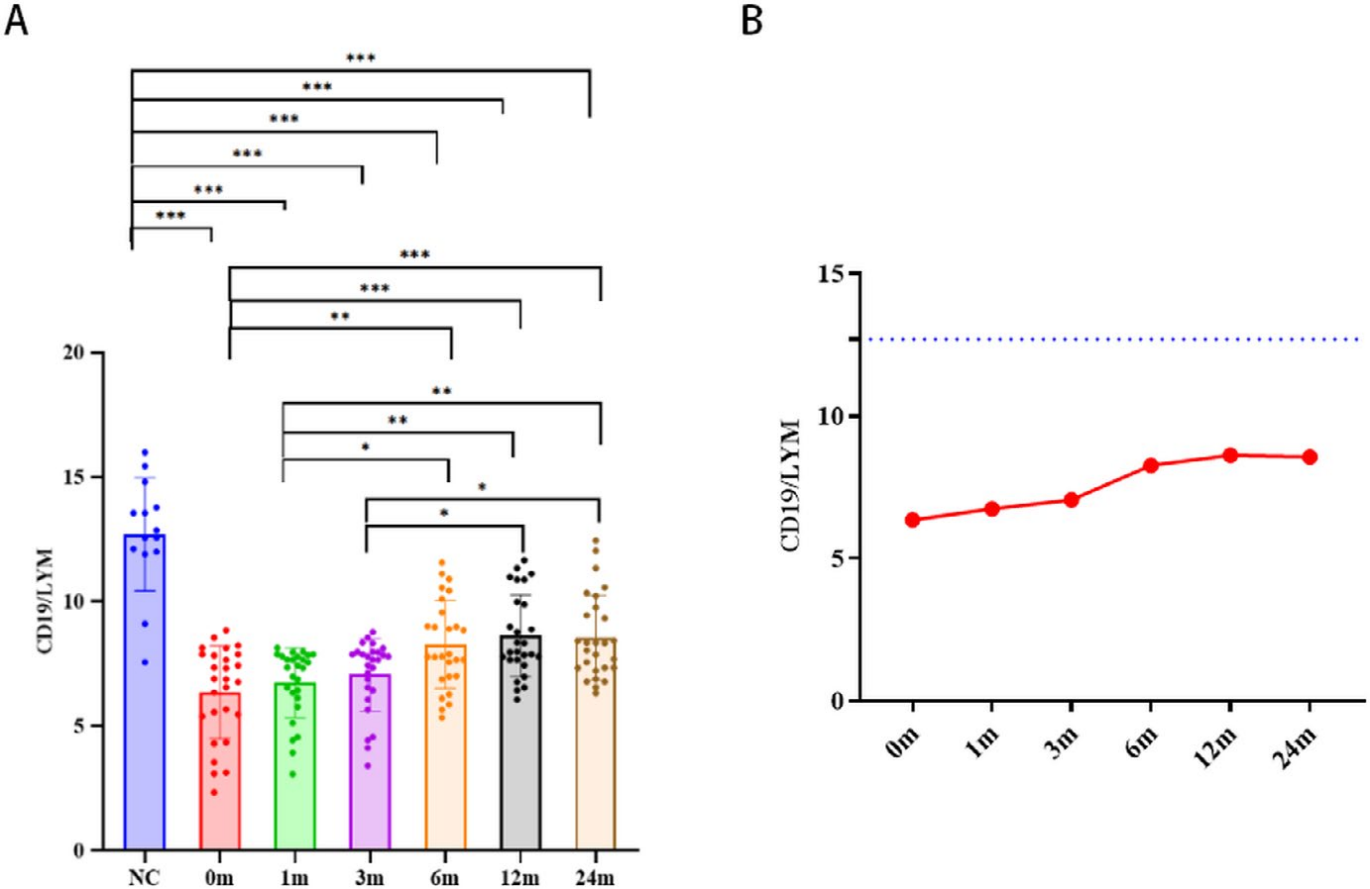
Changes in B lymphocyte proportions in PNH patients receiving C5 complement inhibitor therapy. (A) B lymphocyte proportions and (B) dynamic changes of B lymphocytes from baseline to 24 months of treatment. Data are presented as mean ± SD. *p < 0.05, **p < 0.01, ***p < 0.001.

The proportion of peripheral circulating B cells in PNH patients (6.535 ± 1.854)% was significantly lower than that in the control group (12.70 ± 2.256)% (*p* < 0.001). The number of B cells showed an upward trend after the treatment with complement C5 inhibitor but reached a plateau from 6 to 24 months, remaining below normal control (Figure 3, Table 2).

### 3.5 NK Cells and DCs Did Not Recover After Treatment

**FIGURE 4:**
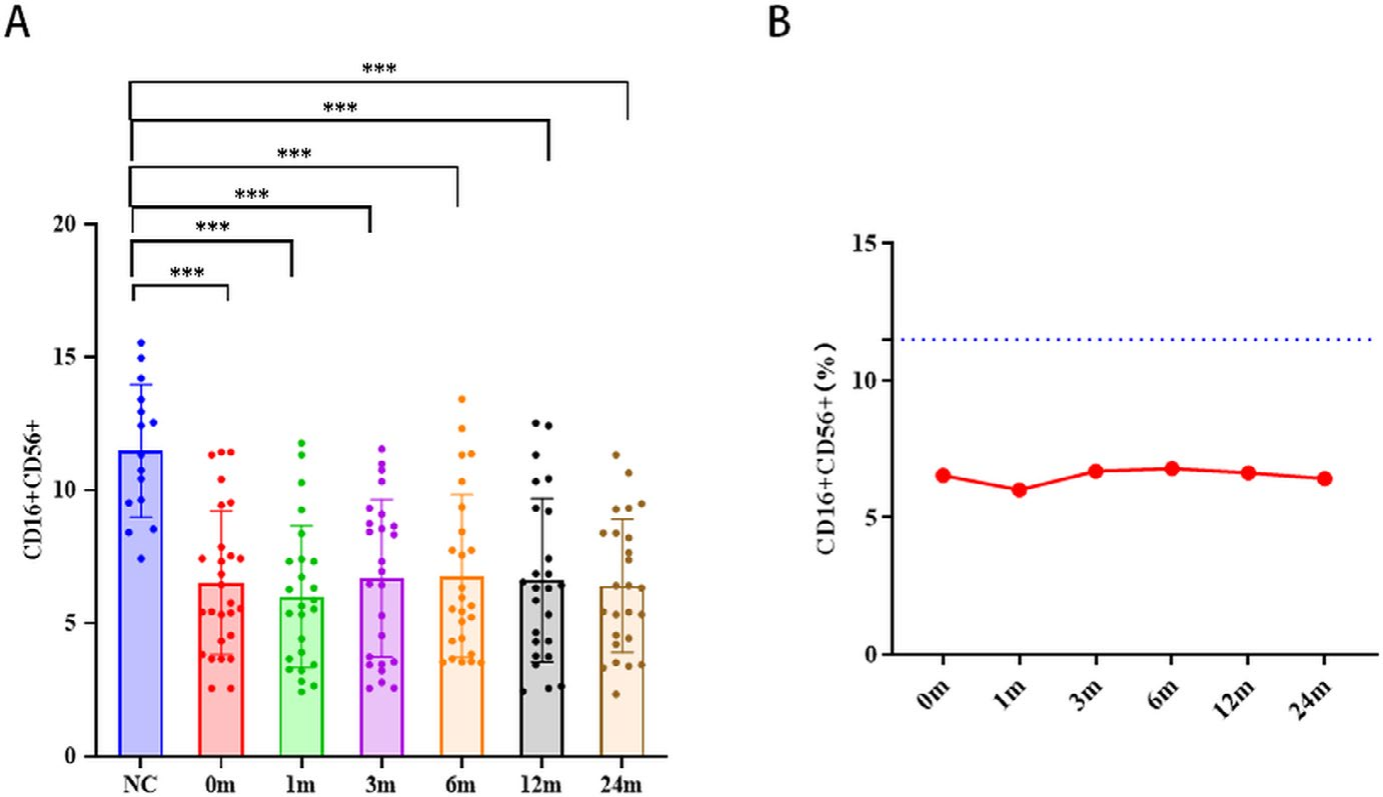
Proportions of NK cells in PNH patients receiving C5 complement inhibitor therapy. (A) NK cell proportions and (B) dynamic changes of NK cells from baseline to 24 months of treatment. Data are presented as mean ± SD. *p < 0.05, **p < 0.01, ***p < 0.001.

The proportion of peripheral circulating NK cells in PNH patients (6.535 ± 2.692)% was significantly lower than that in the control group (11.49 ± 2.500)% (*p* < 0.001). NK cells did not recover after treatment (Figure 4, Table 2).

**FIGURE 5:**
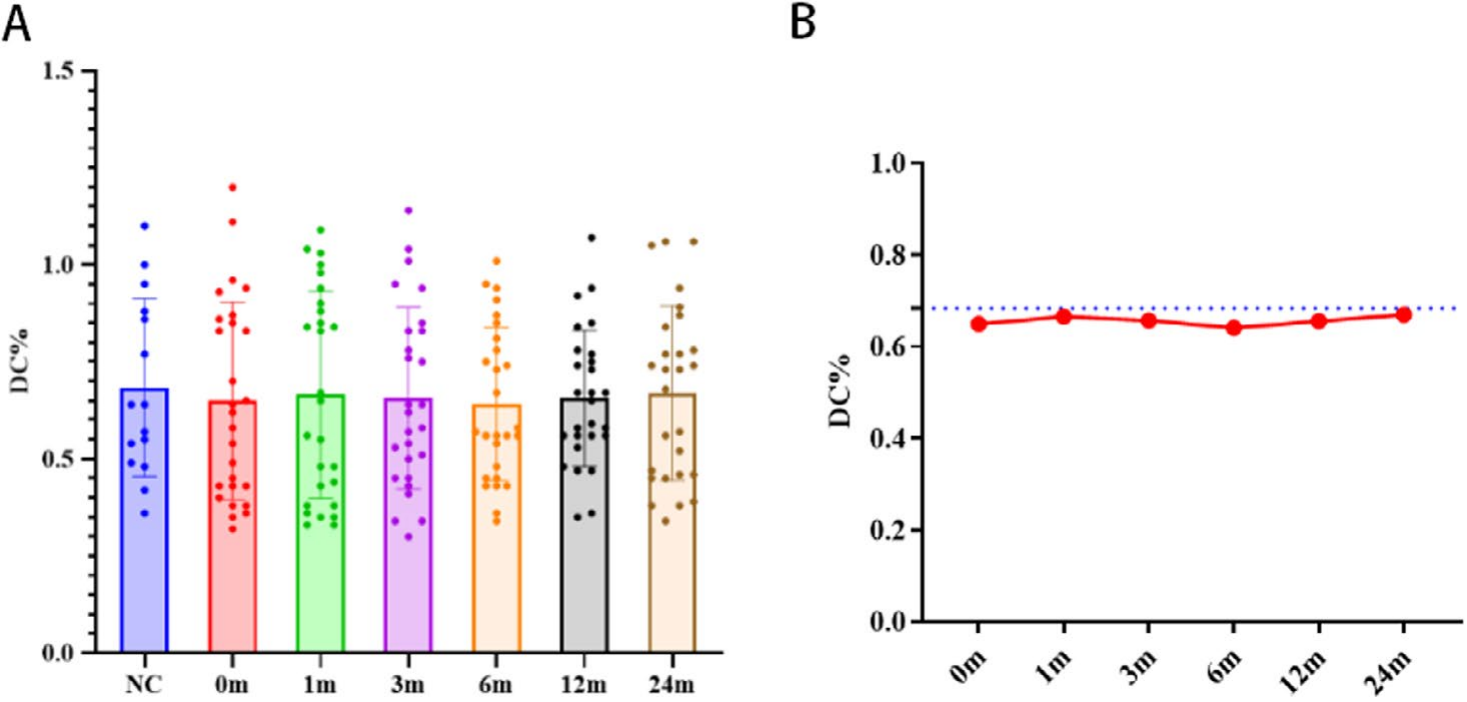
Proportions of DC cells in PNH patients receiving C5 complement inhibitor therapy. (A) DC cell proportions and (B) dynamic changes of DC cells from baseline to 24 months of treatment. Data are presented as mean ± SD. *p < 0.05, **p < 0.01, ***p < 0.001.

There was no statistically significant difference of DC cells between PNH patients (0.62 ± 0.344)% and the control group (0.53 ± 0.265)%. And DCs did not recover after treatment (Figure 5, Table 2).

## 4 Discussion

Paroxysmal nocturnal hemoglobinuria is a disease caused by acquired mutations in the glycosphingolipid‐A (PIG‐A) gene of hematopoietic stem cells. The mutation of the PIG‐A gene leads to the synthesis disorder of glycosylphosphatidylinositol (GPI), resulting in the deficiency of GPI‐anchored proteins (GPI‐AP) on the surface of the cell membrane. As we all know, the possible mechanisms of abnormal clonal proliferation in PNH mainly include immune escape, anti‐apoptosis mechanism, and secondary gene mutation [3, 4, 12, 13, 14].

The immune escape hypothesis suggests that there is an immune attack against hematopoietic stem cells (HSCs) in the pathogenesis of PNH [3, 4, 15]. HSCs with PIG‐A gene mutations and lacking anchor chain proteins GPI‐APs can escape from this immune attack and thus have a survival advantage. To further explore the mechanism of immune escape in PNH, Wang et al. conducted a detailed analysis of the immune status of PNH patients and found an increase in CD8^+^ T cells, a significant decrease in B cells, DC cells, and NK cells [16]. Our team detected changes in bone marrow hematopoietic cells in the patients with PNH by single‐cell sequencing technology and discovered various immune cell abnormalities. We observed a significant increase of T lymphocytes and NK cells, with the main activation of CD59^+^cells, indicating the involvement of the immune escape [17].

T cells play an important role in the proliferation of PNH clone [18]. Identification of PIG‐A mutant T lymphocytes in healthy adults suggests PNH could develop following intense negative selection of hematopoiesis, with clonal outgrowth of naturally occurring PIG‐A mutant stem cells [19]. Ikeda et al. found that WT1 peptide‐specific and HLA‐restricted CTLs may play an important role in the expansion of a PNH clone during immunologic selection and/or in the occurrence of BM failure via interferon‐gamma in PNH [20]. Merchav et al. revealed a pronounced inhibition of primitive erythroid (BFU‐E) progenitor cell growth by marrow T lymphocytes in PNH. They removed T cells prior to culture, resulting in a 4.5‐fold enhancement of BFU‐E numbers [21].

Research suggests that T lymphocytes are involved by two possible mechanisms of immune escape pathogenesis: one is that CD1d MHC presents GPI molecules as antigens to T lymphocytes and activates them, thereby killing GPI + HSCs; however, the MHC class II molecule presented by the GPI‐PNH clone is reduced, allowing the PNH clone to escape this attack; another is that GPI‐AP mediates the activation of T lymphocytes and promotes the release of cytokines such as IL‐2, which then kills normal HSCs expressing GPI‐AP; however, abnormal clones that do not express GPI‐AP can avoid this killing effect [4].

The treatment with eculizumab can significantly control hemolysis and improve clinical symptoms, but there are currently no reports on the recovery of immune status. Our research found that both CD4^+^ and CD8^+^ T cells significantly recovered, approaching the normal range. Novel insights into complement biology have identified cell‐autonomous and intracellularly active complement, the complosome, as an unexpected central orchestrator of normal cell physiology [22]. Intracellular C3 was first discovered in CD4^+^ T cells, and later detected in various tissue cells. Some research has found that complement components can support normal behavior of immune cells through some non‐canonical functions [23, 24, 25].

In Th1 cells, intracellular C3b can promote the secretion of IFNɤ by binding to CD46, while intracellular C5a can lead to the assembly of the NOD, LRR, and pyrin domain‐containing protein 3 inflammasome NLRP3 and the secretion of IL‐1β by binding to mitochondrial C5aR1 [26, 27]. C1q, from the extracellular environment, acts on human and mouse CD8^+^ T cell mitochondria to restrain glycolysis and effector function in the setting of infection to reduce pathological tissue conditions [28]. These also explains why T lymphocytes recover after the application of complement inhibitors.

We also found that the proportion of B cells recovered after the treatment of complement inhibitors but still did not reach normal levels. Early studies of animals bearing natural deficiencies in complement C3 and C4 and mice transiently deficient in C3 suggested that the complement system played a role in humoral immunity. More research shows that C3 and C4 play a role in different stages of B cell differentiation [29]. Paiano et al. show that Ab production and class switch recombination (CSR) depend on autocrine C3a and C5a receptor (C3ar1/C5ar1) signaling in B2 cells [30]. Another research found that Human plasma C3 is essential for the development of memory B cells [31]. Therefore, we speculate that the treatment of C5 inhibitors in patients with PNH may lead to the accumulation of C3, and excessive C3 maybe promote the proliferation and differentiation of B cells to some extent.

NK cells may also play a role in immune escape. NK cells showed no changes before and after complement inhibitor treatment, suggesting that they may regulate immunity through other pathways. El‐Sherbiny et al. found that GPI^−^CD56^bright^ NK cells were significantly more abundant in the patients with PNH, while GPI^+^CD56^bright^ NK cells were not detected [32]. Futher research showed differential chemokine receptor expression between GPI^+^ and GPI^−^ NK cells and impaired stromal cell‐derived factor 1 (SDF‐1)‐induced chemotaxis of GPI^−^ NK cells [33]. Giustiniani reported circulating NK cells from PNH patients, exhibiting a self‐MHC class I molecule reactive effector function, which provided new insights regarding the possible role of CD160‐TM on PNH patients NK lymphocytes [34]. The application of C5 complement inhibitors did not restore NK cells.

In conclusion, our study provides the first systematic analysis of immune cell recovery in PNH patients following C5 complement inhibition. We found that this therapy promotes the recovery of CD4^+^ T, CD8^+^ T, and B cell populations, while Treg, NK, and DC subsets remain largely unchanged. These results suggest that the complement system plays an important role in the immune escape mechanisms of PNH. Clinically, regular monitoring of T and B cell subsets may serve as a useful indicator for treatment response, complementing standard efficacy criteria. Further multi‐center studies with larger patient numbers and longer follow‐up are needed to confirm the stability of T and B cell recovery over time and to examine its relationship with key clinical outcomes, such as disease relapse, thrombotic events, and overall survival. In addition, mechanistic studies are required to clarify how complement inhibition affects T and B cell function in PNH.

## Author Contributions

Rong Fu designed the study and revised the manuscript. Hui Liu and Mengting Che analyzed the data and wrote the manuscript. Liyan Li, Honglei Wang, Chaomeng Wang, Junshu Wu, Yingying Chen, Zhaoyun Liu, and Lijuan Li contributed to patient data collection. All the authors have read and approved the final version of this manuscript.

## Funding

This work was supported by National Natural Science Foundation of China (Grants 82400166, 82270142, 82000128, 81970115, 81770110), the Tianjin Municipal Natural Science Foundation (Grant 24ZGSSSS00050), the Tianjin Science and Technology Planning Project (Grant 24ZXGZSY00090), Tianjin Municipal Health Commission Project (Grant TJWJ2023XK003).

## Ethics Statement

This study was approved by the Ethics Committee of the Tianjin Medical University General Hospital (Ethical no. IRB2025‐YX‐050‐01).

## Conflicts of Interest

The authors declare no conflicts of interest.

## Data Availability

The data that support the findings of this study are available from the corresponding author upon reasonable request.
